# Effect of innate antiviral glycoproteins in breast milk on seroconversion to rotavirus vaccine (Rotarix) in children in Lusaka, Zambia

**DOI:** 10.1371/journal.pone.0189351

**Published:** 2017-12-28

**Authors:** Katayi Mwila-Kazimbaya, Miguel Pugliese Garcia, Samuel Bosomprah, Natasha Makabilo Laban, Caroline Cleopatra Chisenga, Sallie Robey Permar, Michelo Simuyandi, Sody Munsaka, Roma Chilengi

**Affiliations:** 1 Centre for Infectious Disease Research in Zambia, Lusaka, Zambia; 2 Department of biomedical sciences, School of Health sciences, University of Zambia, Lusaka, Zambia; 3 Department of Biostatistics, School of Public Health, University of Ghana, Legon, Accra, Ghana; 4 Department of Paediatrics, Human Vaccine Institute, Duke University, Durham, North Carolina; 5 University of North Carolina at Chapel Hill, School of Medicine, Chapel Hill, North Carolina, United States of America; University of Liverpool, UNITED KINGDOM

## Abstract

**Introduction:**

Rotavirus vaccines have been introduced into national immunization programmes to mitigate morbidity and mortality associated rotavirus diarrhoea. Lower vaccine effectiveness has however been noted in low-middle income countries, but little is known about the role of maternal components found in breast milk. This study assessed the effect of lactoferrin, lactadherin, and tenascin-c on rotavirus vaccine seroconversion.

**Methods:**

This was a retrospective cohort study of 128 infants who had been fully immunized with Rotarix™. Serum samples were collected from the infant at baseline and one month after second rotavirus vaccine dose. Breast milk samples were collected from mothers at baseline. Standard ELISA was used to determine titres of rotavirus-specific immunologlobulin G and A in breast milk and serum as well as concentrations of lactoferrin, lactadherin, and tenascin-c. Poisson regression model with robust standard error was used to estimate the effect of breast milk components on seroconversion. The components were modelled on log base 2 so that the effect would be interpreted as a doubling of the concentration.

**Results:**

In a multivariable analysis adjusting for maternal age, maternal HIV status, seropositivity at baseline, sex, age of child at vaccination as well as breast milk IgA and IgG, we found evidence of independent effect of LA (Adjusted IRR = 0.95; 95% CI = 0.91–0.99; P = 0.019) on seroconversion while there was no evidence for TNC (Adjusted IRR = 1.00; 95% CI = 0.85–1.17; P = 0.967) and LF (Adjusted RR = 1.01; 95% CI = 0.96–1.05); P = 0.802). We explored the joint effects of the three components but we found no evidence (Adjusted RR = 0.95; 95% CI = 0.81; P = 0.535).

**Conclusion:**

High breast milk concentrations of lactadherin might play a role in infant’s failure to seroconvert to rotavirus vaccines. Further research to understand this observed association is an important consideration.

## Introduction

Poor efficacy of orally administered rotavirus vaccines in many low and middle income countries (LMICs) has spurred great efforts towards understanding the reasons behind this observed trend [1–4]. Several factors explaining this reduced efficacy have been postulated including malnutrition, concurrent administration with other vaccines, infection with various enteric pathogens, environmental enteropathy, host genetic and maternal factors [5–14].

Maternal factors affecting rotavirus vaccine efficacy have become a focus of attention with research showing that in addition to immunologic components of breast milk (immunoglobulin A & B, (IgA and IgG)), non-immunologic components may also play a role in reduced vaccine efficacy [15]. Moon and colleagues (2013) showed that women from LMICs had higher rotavirus specific antibodies as well as higher levels of non-antibody breast milk antiviral glycoproteins, including lactoferrin (LF) and lactoadherin (LA), and demonstrated that these non-antibody components contributed to increased neutralization activity of breast milk against rotavirus [15]. Tenascin-C (TNC) has also recently become the focus of research as another non-immunological breast milk component that has shown activity against the HIV virus through possible blocking of the HIV-1 envelope and might therefore contribute to breast milk viral neutralizing properties [16]. Work from Nicaragua failed to show any associations between innate immune factors LF, LA or TNC and seroconversion of infants to pentavalent rotavirus vaccine (RV5) [17]. This difference in results between Moon and colleagues [15] and Becker-Dreps and colleagues [17] shows the need to determine which are key factors in the various settings.

Lactoferrin is a highly multi-functional protein found in secretory fluids such as tears, saliva, nasal secretions and breast milk [18]. It has been well researched for its anti-bacterial, anti-fungal and anti-viral properties [19–21]. Lactadherin is a milk fat protein also noted for its antiviral properties [22], while TNC is a highly conserved extracellular glycoprotein that has been previously associated with fetal growth development and wound healing properties [23, 24].

This study investigated the effect of maternal breast milk levels of TNC, LA and LF in Zambian women on their infant’s seroconversion to the monovalent vaccine Rotarix™ (GlaxoSmithKline Biologicals, Rixensart, Belgium) when routinely immunized within the expanded programme on immunization in Zambia.

## Materials and methods

The study protocol was approved by the University of Zambia Biomedical Research Ethics Committee, University of North Carolina at Chapel Hill Institutional Review Board and the Zambian Ministry of Health. The study was conducted in accordance with the principles of the Declaration of Helsinki and in compliance with good clinical practice guidelines; ClinicalTrials.gov registration number NCT 01886833.

### Study site and participants

The study site and participant enrolment was as described previously [25]. Briefly, a population of eligible and consenting mothers and their infants attending routine immunization at Kamwala Clinic in Lusaka Zambia were recruited into the study. At 6 weeks post-partum (baseline), a sample of breast milk was provided by the mother through manual expression and immediately stored at -80°C. Blood was drawn from their infants at the time of breast milk collection for measurement of rotavirus-specific IgA and IgG described in detail previously [25] after which the infants received the routinely scheduled immunizations including rotavirus vaccine, Rotarix™. A second blood draw was taken from infants one month after receiving the second dose of Rotarix™ to measure rotavirus-specific IgA which was used to determine the seroconversion status of the infants.

### Measurement of LF, LA and TNC in breast milk

Enzyme-linked immunosorbent assay (ELISA) was used for measurement of LF, LA and TNC in breast milk samples. LF concentrations were determined using commercial Human Lactoferrin (HLF2) ELISA kit (ab108882 Abcam, Cambridge United Kingdom) according to the manufacturer’s instructions except that the aqueous portion of breast milk samples was serially diluted to 1:1,000,000. Briefly, breast milk samples were centrifuged at 800 x *g* for 10 minutes and the diluted aqueous portion added to microplate wells pre-coated with LF antibody and incubated. After washing, wells were treated with human LF-specific biotinylated detection antibody, followed by another wash step and incubated with streptavidin peroxidase conjugate. After incubation, color reactions were developed with chromogen substrate, 3,3’,5,5’-tetramethylbenzidine, and stopped with stop solution. Optical density was read at 450nm and 570nm for wavelength correction and LF concentrations were determined using assay standard curve. Commercial ELISA kits were similarly used to determine concentrations of LA (LS-F12031, LifeSpan BioSciences USA) and TNC (LS-F22079, LifeSpan BioSciences USA). Samples were centrifuged at 800 x g for 10 minutes, LA samples were left undiluted and TNC diluted 1:100 and assayed as with LF substituting Avidin-Horseradish Peroxidase during color development. Optical density was read at 450nm wavelength and concentrations were determined based on the assay standard curve.

### Post-hoc power calculation

We calculated the post-hoc power for a given sample size of 128 available for this study. For a 40% prevalence of non-seroconverted infants one month following full rotavirus immunisation, this study is powered at about 70% to detect a decrease to 20% in the proportion of babies not seroconverted using a two-sided Pearson’s chi-squared test at 5% level of significance.

### Statistical analysis

The primary outcome was vaccine seroconversion in infants defined as a four-fold or greater increase in rotavirus-specific IgA titre between baseline and 1 month post Rotarix™ second dose. IgA titre higher or equal to 40 in serum was considered positive. IgA titre below limit of detection (BLD) (i.e. smaller than 20) in serum was assigned a value of 1 [8, 25, 26]. Breast milk glycoproteins (TNC, LA and LF), breast milk rotavirus specific-IgA, and infant serum rotavirus specific-IgG were categorized as quartiles and we used Wilcoxon rank-sum test for trend to examine association with seroconversion. For other infant and maternal factors, we used Pearson’s Chi-squared test to examine associations. Spearman’s correlation coefficient was used to examine correlation between the breast milk antiviral glycoproteins. Where there was no evidence of pairwise correlation, we included all the components in the regression model to assess independent effects.

Poisson regression model with robust standard error was used to estimate the independent effects of maternal breast milk levels of TNC, LA and LF on seroconversion adjusting for infant and maternal factors. The maternal breast milk levels of TNC, LA and LF were transformed into log base 2 scales and modelled as a continuous covariate so that the effect would be interpreted as a doubling of the concentration. Zero concentrations were imputed with a very small value (i.e. 0.01) before log transformation so that we do not set them to missing. In the multivariable model, we estimated the independent as well as joint effects of the maternal breast milk levels of TNC, LA and LF on seroconversion. Covariates (i.e. breast milk, maternal and child characteristics) were retained in the model regardless of their p-values to improve the precision of the estimates. In a sensitivity analysis, we explored the influence of BLD by assigning half the smallest titre (i.e. 10). Data were analysed using Stata 14 (StataCorp, College Station, Texas, USA).

## Results

### Characteristics of participants

**Fig 1:**
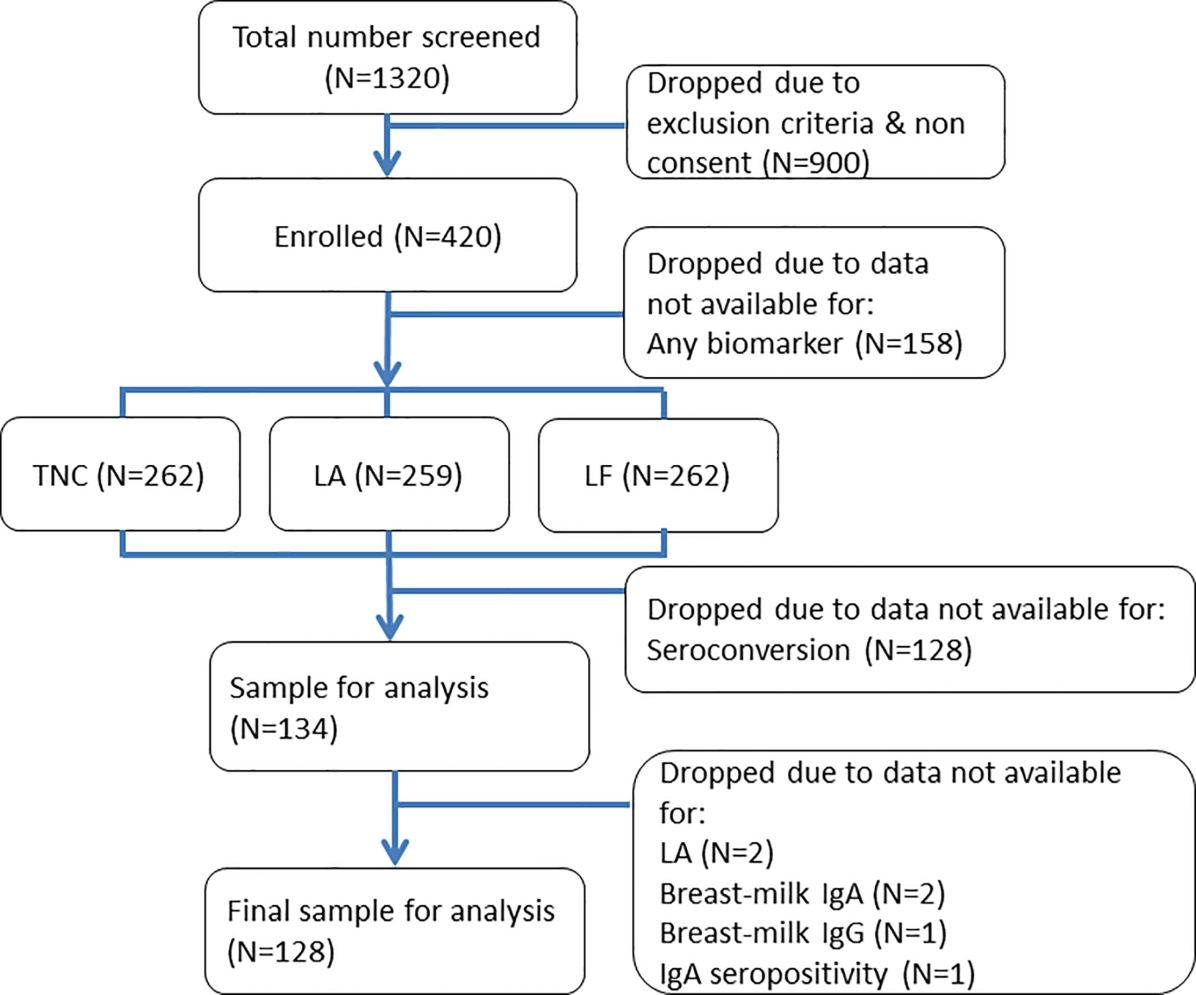
Study participants flow chart.

A total of 420 mother-infant pairs successfully enrolled in the primary study. Information on non-immunological components was collected from 262 and information on seroconversion from 134. This was partly due to participants’ failure to bring infants for post vaccination blood draws and drop out after initial enrolment. After removing 6 observations with incomplete data for one or more of the variables in our analysis, the final sample for analysis comprised a total of 128 individuals with full data; see **Fig 1.** The median breast-milk anti-rotavirus IgA was 160 (Interquartile range [IQR] = 80–320) while the median breast-milk anti-rotavirus IgG was 5,120 (IQR = 2,560–10,240) (Table 1).

**Table 1:** Frequency of seroconversion after second vaccine dose by breast milk components and key infant and maternal factors among rotavirus-vaccinated infants aged 6–11 weeks. Values below limit of detection were imputted as 1.

Breat-milk component	Number of infants (% of total)	No. (%) of seroconverted	95% CI	P-Value^1^
**TNC—Quartiles (median conc. (mg/ml))**				
Median (IQR)	1.09 (0.77–36.00)		-	-
1 (0.38)	30 (23)	18 (60)	(41–76)	0.564
2 (0.92)	29 (23)	19 (66)	(46–81)	
3 (1.21)	36 (28)	21 (58)	(42–73)	
4 (1.64)	33 (26)	23 (70)	(52–83)	
**LA—Quartiles (median conc. (pg/ml))**				
Median (IQR)	0 (0–54.43)		-	-
1+2 (1)	67 (52)	50 (75)	(63–84)	**0.006**
2 (20)	30 (23)	16 (53)	(35–70)	
3 (280)	31 (24)	15 (48)	(31–66)	
**LF—Quartiles (median conc. (mg/ml))**				
Median (IQR)	60,538 (20,254–74,530)		-	-
1+2 (13,745)	31 (24)	20 (65)	(46–79)	0.577
2 (39,628)	28 (22)	15 (54)	(35–71)	
3 (74,062)	69 (54)	46 (67)	(55–77)	
**Breast-milk anti-rotavirus IgA—Quartiles (median titre)**				
Median (IQR)	160 (80–320)		-	-
1 (40)	60 (47)	44 (73)	(61–83)	**0.008**
2 (160)	25 (20)	17 (68)	(47–83)	
3 (320)	21 (16)	10 (48)	(27–69)	
4 (960)	22 (17)	10 (45)	(26–66)	
**Breast-milk anti-rotavirus IgG—Quartiles (median titre)**				
Median (IQR)	5,120 (2,560–10,240)		-	-
1 (1,280)	45 (35)	34 (76)	(61–86)	0.095
2 (5,120)	26 (20)	15 (58)	(38–75)	
3 (10,240)	31 (24)	16 (52)	(34–69)	
4 (20,480)	26 (20)	16 (62)	(42–78)	
**Sex of child**				
Female	72 (56)	44 (61)	(49–72)	0.564
Male	56 (44)	37 (66)	(53–77)	
**Age of child at vaccination (Weeks)**				
<7	78 (61)	52 (67)	(55–76)	0.321
7+	50 (39)	29 (58)	(44–71)	
**Seropositivity at baseline**				
No	100 (78)	65 (65)	(55–74)	0.446
Yes	28 (22)	16 (57)	(31–74)	
**Maternal age**				
16–19	21 (16)	13 (62)	(40–80)	0.695
20–29	83 (65)	51 (61)	(50–71)	
30–39	24 (19)	17 (71)	(49–86)	
**Maternal HIV Status**				
Negative	85 (66)	59 (69)	(59–78)	**0.043**
Positive	43 (34)	22 (51)	(36–66)	
**Season of vaccination**				
Dry (May-October)	76 (59)	49 (64)	(53–75)	0.735
Wet (November-April)	52 (41)	32 (62)	(48–74)	
**Total**	128	81 (63)	(54–71)	

^1^ Wilcoxon rank-sum test for trend

^2^ Chi-square test

The median breast milk TNC, LA, and LF titres for the entire sample were 1.09 mg/ml (IQR = 0.77–36.00), 0 pg/ml (IQR = 0–54.43) and 60,538 mg/ml (IQR = 20,254–74,530) respectively (Table 1). Overall, 63% (81/128) of infants seroconverted following rotavirus vaccination (Table 1). Infants whose mothers had higher titres of LA (P = 0.006) were less likely to seroconvert (Table 1).

### Breast milk components independently associated with seroconversion

In univariable analyses, we found evidence of an association between LA and seroconversion (Crude IRR **=** 0.94; 95% CI = 0.90–0.99; P = 0.009) while there was no evidence of association between TNC, LF and seroconversion (Table 2). In a multivariable analysis adjusting for maternal age, maternal HIV status, seropositivity at baseline, sex, age of child at vaccination as well as breast milk IgA and IgG we found evidence of independent effect of LA (Adjusted IRR = 0.95; 95% CI = 0.91–0.99; P = 0.019) on seroconversion while there was no evidence for TNC (Adjusted IRR = 1.00; 95% CI = 0.85–1.17; P = 0.967) and LF (Adjusted RR = 1.01; 95% CI = 0.96–1.05); P = 0.802) (Table 2). We explored the joint effects of the three non-immunologic factors but we found no evidence (Adjusted RR = 0.95; 95% CI = 0.- 0.81; P = 0.535) (Table 2). We didn’t find any correlations between the breast milk component concentrations.

**Table 2:** Effects of non-immunological factors in breast milk on vaccine seroconversion post second dose among rotavirus-vaccinated infants aged 6–11 weeks. Values below limit of detection were imputed to 1.

Biomarker	IRR (95%CI)	P-value	Adjusted RR (95%CI) ^1^	Adjusted P-value
**TNC**				
	1.05 (0.89, 1.24)	0.546	1.00 (0.85, 1.17)	0.967
**LA**				
	0.94 (0.90, 0.99)	**0.009**	0.95 (0.91, 0.99)	**0.019**
**LF**				
	1.01 (0.96, 1.05)	0.8	1.01 (0.96, 1.05)	0.802
**Joint effect TNC, LA, LF**^2^				
	1.01 (0.85, 1.19)	0.947	0.95 (0.81, 1.12)	0.535

^1^ Estimates were adjusted for Breast-milk anti-rotavirus IgA (transformed on log base 2); Infant serum anti-rotavirus IgG titre (transformed on log base 2); Maternal age (categorical); Maternal HIV status; Seropositivity at baseline (IgA > = 1:40) (binary); Age of child at vaccination (binary); Sex and TNC, LA and LF (transformed on log base

^2^ Joint effect was calculated using linear combination of TNC, LA and LF and estimated using the lincom command

### Sensitivity analysis

In a sensitivity analysis to explore the effect of handling values BLD on the case definition of seroconversion, we found that when imputed by half the smallest titre (10), instead of by one, the independent effects of LA, TNC, and LF were similar except that the 95% CIs were slightly wider (Table 3), suggesting that our study is underpowered to detect such effect.

**Table 3:** Effects of non-immunological factors in breast milk on vaccine seroconversion post second dose among rotavirus-vaccinated infants aged 6–11 weeks. Values below limit of detection were imputed to half the lowest titre in that particular component for sensitivity purposes.

Biomarker	IRR (95%CI)	P-value	Adjusted RR (95%CI) ^1^	Adjusted P-value
**TNC**				
	1.00 (0.84, 1.20)	0.974	0.94 (0.78, 1.12)	0.473
**LA**				
	0.96 (0.92, 1.00)	0.057	0.97 (0.93, 1.02)	0.214
**LF**				
	1.03 (0.91, 1.17)	0.657	1.02 (0.91, 1.15)	0.686
**Joint effect TNC, LA, LF** ^2^				
	0.99 (0.81, 1.21)	0.914	0.93 (0.77, 1.14)	0.502

^1^ Estimates were adjusted for Breast-milk anti-rotavirus IgA (transformed on log base 2); Infant serum anti-rotavirus IgG titre (transformed on log base 2); Maternal age (categorical); Maternal HIV status; Seropositivity at baseline (IgA > = 1:40) (binary); Age of child at vaccination (binary); Sex and TNC, LA and LF (transformed on log base

^2^ Joint effect was calculated using linear combination of TNC, LA and LF and estimated using the lincom command

## Discussion

We found that increasing concentration of maternal breast milk LA in Zambian women might play a role on their infant’s inability to seroconvert to the monovalent vaccine Rotarix™ (GlaxoSmithKline Biologicals, Rixensart, Belgium) when routinely immunized within the expanded programme on immunization in Zambia. However, there was no evidence from our study regarding the role of TNC or LF or jointly (TNC, LF, LA) on seroconversion.

Very little is known about the association of breast milk components with rotavirus vaccine response. In the only such study conducted in Nicaragua, Becker-Dreps and colleagues failed to find association between seroconversion and innate immune factors (Lactoferrin, Lactadherin, Tenascin-C) in mother’s breast milk [17]. The observed association of LA with seroconversion found in our study is interesting. In our study, we observed that over half of the mothers have zero LA concentrations (median = 0 pg/ml (IQR = 0–54.43), possibly pointing to the fact that even small differences in concentration could have a sizeable effect. This tallies with the conclusion reached by Newburg and colleagues (1998) who noted that higher LA concentrations were able to play a key role in mitigating rotavirus symptomatic infection even in the absence of LF [22]. The conclusion by Moon and colleagues [15] found that increased LF and LA concentrations was associated with an increase in virus neutralization activity, which would point towards our finding of LA neutralizing the vaccine and thus impacting on vaccine uptake. Our study failed to find any correlations between TNC, LA and LF. This could be explained by the differences in location of the molecules and the genetic make up. Newburg and collegues (1998) showed that LA was a milk fat globule (MFG) protein located in the hydrophobic membrane with a heterogeneous sugar backbone that is determined by the Lewis blood group and secretor status. Lewis status deterimined differences in the carbohydrate moiety that could affect its expression and ultimately concentrations found in milk [22]. Whereas TNC and LF are secretory hydrophilic components and these differences in properties would not allow for synergism between the components hence the lack of association observed.

Although our results showed that the presence of TNC, LA, and LF together did not have an effect on rotavirus vaccine seroconversion among the infants, further work is needed before a conclusion can be made. An analysis of this nature may clarify the work by Moon et al (36) who noted that as concentrations of LF and LA increased so did rotavirus neutralization. Given that nearly all mothers had detectable TNC and LF levels in breast milk, this could be a limiting factor in analysis of the independent effect of LA as it could have been masked behind the total effect of all three non-immunological factors. Thus, further research on the effect of LA and its ability to affect rotavirus seroconversion in infants in the absence of the other two components may be of interest.

Our analysis also showed that fewer children of HIV positive mothers seroconverted than those of uninfected mothers (P = 0.035) which did not remain significant after adjusting for LA, suggesting potential interference by HIV exposure with infant’s response to rotavirus vaccine. The result of HIV exposure being significant is of interest to us. Despite literature showing that infants are able to mount adequate immune responses to rotavirus vaccine regardless of their HIV exposure status [27–29], it once again reminds us that HIV exposed children present a challenging population subset that require careful understanding of its potential interference with immune response to rotavirus vaccine. Our data suggests that the observed level of LA could have some association with HIV exposure status of children. This however is another area for further research with regards to the mode of actions of these factors on rotavirus vaccines in the context of HIV.

Another area of consideration for further research is the handling of values BLD for the case definition of seroconversion. The use of 1 to impute sBLD is conventional but a methodological study to examine optimum values to impute for BLD is an important consideration.

## Conclusions

Our study found association of LA (but not TNC and LF) with seroconversion. There is need for further work to determine if breast milk concentrations LA and rotavirus IgA contribute to the lower seroconversion rate in infants after rotavirus vaccination in developing regions.

## References

[1] Madhi SA, Cunliffe NA, Steele D, Witte D, Kirsten M, Louw C, et al Effect of human rotavirus vaccine on severe diarrhea in African infants. N Engl J Med. 2010;362(4):289–98. doi: 10.1056/NEJMoa0904797 20107214 10.1056/NEJMoa0904797

[2] Ruiz-Palacios GM, Pérez-Schael I, Velázquez FR, Abate H, Breuer T, Clemens SC, et al Safety and efficacy of an attenuated vaccine against severe rotavirus gastroenteritis. N Engl J Med. 2006;354(1):11–22. doi: 10.1056/NEJMoa052434 16394298 10.1056/NEJMoa052434

[3] Vesikari T, Karvonen A, Puustinen L, Zeng S-Q, Szakal ED, Delem A, et al Efficacy of RIX4414 Live attenuated human rotavirus vaccine in Finnish infants. Pediatr Infect Dis J. 2004;23:10. 10.1097/01.inf.0000141722.10130.50 15602194

[4] Zaman K, Dang DA, Victor JC, Shin S, Yunus M, Dallas MJ, et al Efficacy of pentavalent rotavirus vaccine against severe rotavirus gastroenteritis in infants in developing countries in Asia: a randomised, double-blind, placebo-controlled trial. Lancet. 2010;376(9741):615–23. doi: 10.1016/S0140-6736(10)60755-6 20692031 10.1016/S0140-6736(10)60755-6

[5] Gilmartin AA, Petri WAJ. Exploring the role of environmental enteropathy in malnutrition, infant development and oral vaccine response. Philosophical transactions of the Royal Society of London Series B, Biological sciences. 2015;370(1671). 10.1098/rstb.2014.0143 PMC4527388 25964455

[6] Lopman BA, Pitzer VE, Sarkar R, Gladstone B, Patel M, Glasser J, et al Understanding reduced rotavirus vaccine efficacy in low socio-economic settings. PloS one. 2012;7(8). 10.1371/journal.pone.0041720 PMC3412858 22879893

[7] Moon S, Groome MJ, Velasquez DE, Parashar UD, Jones S, Koen A, et al Prevaccination rotavirus serum IgG and IgA are associated with lower immunogenicity of live, oral human rotavirus vaccine in South African infants. Clin Infect Dis. 2016;62(2):157–65. doi: 10.1093/cid/civ828 26400993 10.1093/cid/civ828

[8] Moon S, Yuhuan WM, Shane AL, Nguyen T, Ray P, Dennehy P, et al Inhibitory effect of breast milk in infectivity of live oral rotavirus vaccines. Pediatr Infect Dis J. 2010;29(10):919–23. doi: 10.1097/INF.0b013e3181e232ea 20442687 10.1097/INF.0b013e3181e232ea PMC3704726

[9] Nakaya HI, Bruna-romero O. Is the gut microbiome key to modulating vaccine?. Expert Rev Vaccines. 2015;14(6):777–9. doi: 10.1586/14760584.2015.1040395 25915555 10.1586/14760584.2015.1040395

[10] Naylor C, Lu M, Haque R, Mondal D, Buonomo E, Nayak U, et al EBioMedicine Environmental Enteropathy, Oral Vaccine Failure and Growth Faltering in Infants in Bangladesh. EBIOM. 2015;2(11):1759–66. 10.1016/j.ebiom.2015.09.036 PMC4740306 26870801

[11] Nordgren J, Sharma S, Bucardo F, Nasir W, Günaydın G, Ouermi D, et al Both Lewis and secretor status mediate susceptibility to rotavirus infections in a rotavirus genotype-dependent manner. Clin Infect Dis. 2014;59(11):1567–73. doi: 10.1093/cid/ciu633 25097083 10.1093/cid/ciu633 PMC4650770

[12] Petri WA, Miller M, Binder HJ, Levine MM, Dillingham R, Guerrant RL. Enteric infections, diarrhea, and their impact on function and development. J Clin Invest. 2008;118(4):1277–90. doi: 10.1172/JCI34005 18382740 10.1172/JCI34005 PMC2276781

[13] Prendergast AJ. Malnutrition and vaccination in developing countries. Philos Trans R Soc B Biol Sci. 2015;370(1671):20140141. 10.1098/rstb.2014.0141 PMC4527386 25964453

[14] Taniuchi M, Platts-Mills JA, Begum S, Uddin MJ, Sobuz SU, Liu J, et al Impact of enterovirus and other enteric pathogens on oral polio and rotavirus vaccine performance in Bangladeshi infants. Vaccine. 2016;34(27):3068–75. doi: 10.1016/j.vaccine.2016.04.080 27154394 10.1016/j.vaccine.2016.04.080 PMC4912219

[15] Moon S, Ray P, Dennehy P, Glass RI. Differential profiles and inhibitory effect on rotavirus vaccines of nonantibody components in breast milk from mothers in developing and developed countries. Pediatr Infect Dis J. 2013;32:863–70. doi: 10.1097/INF.0b013e318290646d 23584581 10.1097/INF.0b013e318290646d PMC4610365

[16] Fouda GG, Jaeger FH, Amos JD, Ho C, Kunz EL, Anasti K, et al Tenascin C is an innate broad spectrum HIV-1 –neutralizing protein in breast milk. PNAS. 2013;110(45):18220–5. doi: 10.1073/pnas.1307336110 24145401 10.1073/pnas.1307336110 PMC3831436

[17] Becker-dreps S, Choi WS, Stamper L, Vilchez S, Velasquez DE, Moon S. Innate immune factors in mothers breast milk and their lack of association with rotavirus vaccine immunogenicity in Nicaraguan infants. J Pediatr Infect Dis Soc. 2015:1–4. 10.1093/jpids/piv076 PMC5907878 26582774

[18] Sánchez L, Calvo M, Brock JH. Biological role of lactoferrin. Arch Dis Child. 1992;67(5):657–61. 1599309 10.1136/adc.67.5.657 PMC1793702

[19] Harmsen MC, Swart PJ, Béthune M-P, Pauwels R, Clercq E, The TB, et al Antiviral Effects of Plasma and Milk Proteins: Lactoferrin Shows Potent Activity against Both Human Immunodeficiency Virus and Human Cytomegalovirus Replication In Vitro. The Journal of infectious diseases. 1995;172(2):380–8. 7622881 10.1093/infdis/172.2.380

[20] Nozaki A, Ikeda M, Naganuma A, Nakamura T, Inudoh M, Tanaka K, et al Identification of a Lactoferrin-derived Peptide Possessing Binding Activity to Hepatitis C Virus E2 Envelope Protein. The Journal of biological chemistry. 2003;278(12):10162–73. doi: 10.1074/jbc.M207879200 12522210 10.1074/jbc.M207879200

[21] Puddu P, Borghi P, Gessani S, Valenti P, Belardelli F, Seganti L. Antiviral effect of bovine lactoferrin saturated with metal ions on early steps of human immunodeficiency virus type 1 infection. Int J Biochem Cell Biol. 1998;30(9):1055–62. 9785469 10.1016/s1357-2725(98)00066-1

[22] Newburg DS, Peterson JA, Ruiz-Palacios GM, Matson DO, Morrow AL, Shults J, et al Role of human-milk lactadherin in protection against symptomatic rotavirus infection. Lancet. 1998;351(9110):1160–4. 9643686 10.1016/s0140-6736(97)10322-1

[23] Imanaka-Yoshida K, Aoki H. Tenascin-C and mechanotransduction in the development and diseases of cardiovascular system. Front Physiol Frontiers Media SA. 2014;5:283. 10.3389/fphys.2014.00283 PMC4114189 25120494

[24] Midwood KS, Orend G. The role of tenascin-C in tissue injury and tumorigenesis. J Cell Commun Signal. 2009;3(3–4):287–310. doi: 10.1007/s12079-009-0075-1 19838819 10.1007/s12079-009-0075-1 PMC2778592

[25] Chilengi R, Simuyandi M, Beach L, Mwila K, Becker-dreps S, Emperador DM, et al Association of maternal immunity with rotavirus vaccine immunogenicity in Zambian infants. PloS one. 2016;11(3):1–12. 10.1371/journal.pone.0150100 PMC4790930 26974432

[26] Groome MJ, Moon S-S, Velasquez D, Jones S, Koen A, van Niekerk N, et al Effect of breastfeeding on immunogenicity of oral live-attenuated human rotavirus vaccine: a randomized trial in HIV-uninfected infants in Soweto, South Africa. Bulletin of the World Health Organization. 2014;92(4):238–45. doi: 10.2471/BLT.13.128066 24700991 10.2471/BLT.13.128066 PMC3967577

[27] Groome MJ, Madhi SA. Five-year cohort study on the burden of hospitalisation for acute diarrhoeal disease in African HIV-infected and HIV-uninfected children: potential benefits of rotavirus vaccine. Vaccine. 2012;30(Suppl 1):A173–8. 22520128 10.1016/j.vaccine.2011.08.004

[28] Obaro SK, Pugatch D, Luzuriaga K. Immunogenicity and efficacy of childhood vaccines in HIV-1-infected children. Lancet Infect Dis 2004;4(8):510–8. doi: 10.1016/S1473-3099(04)01106-5 15288824 10.1016/S1473-3099(04)01106-5

[29] Steele AD, Madhi SA, Louw CE, Bos P, Tumbo JM, Werner CM, et al Safety, reactogenicity, and immunogenicity of human rotavirus vaccine RIX4414 in human immunodeficiency virus-positive infants in South Africa. Pediatr Infect Dis J. 2011;30(2). 10.1097/INF.0b013e3181f42db9 20842070

